# Developing a Digital Solution for Remote Assessment in Multiple Sclerosis: From Concept to Software as a Medical Device

**DOI:** 10.3390/brainsci11091247

**Published:** 2021-09-21

**Authors:** Anneke van der Walt, Helmut Butzkueven, Robert K. Shin, Luciana Midaglia, Luca Capezzuto, Michael Lindemann, Geraint Davies, Lesley M. Butler, Cristina Costantino, Xavier Montalban

**Affiliations:** 1 Department of Neuroscience, Central Clinical School, Monash University, Melbourne, VIC 3004, Australia; helmut.butzkueven@monash.edu; 2 The Alfred, Melbourne, VIC 3004, Australia; 3 MedStar Georgetown University Hospital, Washington, DC 20007, USA; Robert.K.Shin@gunet.georgetown.edu; 4 Servei de Neurologia-Neuroimmunologia, Centre d’Esclerosi Múltiple de Catalunya (Cemcat), Institut de Recerca Vall d’Hebron (VHIR), Hospital Universitari Vall d’Hebron, Universitat Autònoma de Barcelona, 08035 Barcelona, Spain; lmidaglia@cem-cat.org; 5 F. Hoffmann-La Roche Ltd., 4070 Basel, Switzerland; luca.capezzuto@roche.com (L.C.); michael.lindemann@roche.com (M.L.); geraint.davies.gd1@roche.com (G.D.); lesley.butler@roche.com (L.M.B.); costantino.cristina@gene.com (C.C.); 6 Multiple Sclerosis Centre of Catalonia (Cemcat), Department of Neurology/Neuroimmunology, Hospital Universitari Vall d’Hebron, Universitat Autònoma de Barcelona, 08035 Barcelona, Spain; xavier.montalban@cem-cat.org

**Keywords:** multiple sclerosis, software as a medical device, digital health, participatory health, monitoring, smartphone-based assessments, clinical validation, technical validation, MS apps, digital health solution development

## Abstract

There is increasing interest in the development and deployment of digital solutions to improve patient care and facilitate monitoring in medical practice, e.g., by remote observation of disease symptoms in the patients’ home environment. Digital health solutions today range from non-regulated wellness applications and research-grade exploratory instruments to regulated software as a medical device (SaMD). This paper discusses the considerations and complexities in developing innovative, effective, and validated SaMD for multiple sclerosis (MS). The development of SaMD requires a formalised approach (design control), inclusive of technical verification and analytical validation to ensure reliability. SaMD must be clinically evaluated, characterised for benefit and risk, and must conform to regulatory requirements associated with device classification. Cybersecurity and data privacy are also critical. Careful consideration of patient and provider needs throughout the design and testing process help developers overcome challenges of adoption in medical practice. Here, we explore the development pathway for SaMD in MS, leveraging experiences from the development of Floodlight™ MS, a continually evolving bundled solution of SaMD for remote functional assessment of MS. The development process will be charted while reflecting on common challenges in the digital space, with a view to providing insights for future developers.

## 1. Introduction

Multiple sclerosis (MS) is an inflammatory demyelinating and degenerative disease [1] characterised by a wide clinical variability in disease trajectory between individuals [2]. Clinical monitoring is intermittently, and often inconsistently [3,4], applied via in-clinic measures, such as the Expanded Disability Status Scale (EDSS) [5] and magnetic resonance imaging; detecting early disease progression is thus challenging [3,4]. Progressive worsening in specific domains (e.g., cognition [6]) can be subtle or subclinical, especially in the early stages of the disease, but tends to increase in frequency and severity over time. The worsening of disability is a multidimensional process and difficult to detect [7,8]. At present, the diagnosis of progression in MS is typically retrospective with a heavy reliance on clinical history, requiring progressive worsening for more than 6 months based on EDSS score, without evidence of relapses [3,4]. Monitoring in MS relies on infrequent outpatient assessments (typically occurring once or twice annually) with a lack of objective assessments of progression available to healthcare professionals (HCPs). New clinical and research tools are therefore needed to address the unmet need of early detection and ongoing assessment of progressive worsening, rendering this an inviting area for innovation in the digital health space.

Remote digital solutions such as smartphone-based apps, wearables, and decision support algorithms are increasingly utilised in research and clinical trial settings [9] and are beginning to emerge in routine medical care. This paper will focus on smartphone technology, which is ubiquitous and broadly accessible [10,11], making it a viable approach for facilitating remote assessment [12,13,14]. Smartphones can be used in a patients’ home environment as frequently as required and their use is increasingly familiar and unobtrusive. Further, most off-the-shelf smartphones contain sensors with the capacity to gather objective data unaffected by inter- and intra-rater variability. Measurements and patient-reported information captured with smartphone technology have the potential to enable more frequent, decentralised, and home-based care to supplement the infrequent in-clinic assessments typically offered to patients. Mutually sharing this information with patients can help focus the clinical conversation or empower shared decision making. Smartphone-based digital solutions are thus ideally placed to contribute to improving clinical care management for people living with MS (PLwMS) [15,16] and providing personalised healthcare [17].

Today, smartphone applications, performing a variety of functions, are available to support PLwMS. Many of these tools are non-regulated wellness applications designed to support day-to-day disease management, for example through symptom or medication intake tracking, visit-scheduling, provision of disease education, and connectivity to supportive care facilities or patient social media networks [18,19,20,21,22,23]. Other smartphone-based solutions enable assessment of functional parameters affected by the disease, such as mobility and cognition, or therapeutic benefit, such as for fatigue or depression [24]. Data and digital biomarkers collected by patient-facing apps may provide clinical value by generating new insights into the MS disease course, ultimately improving the understanding of individual disease trajectories and response to intervention. Despite their promise, however, smartphone-based solutions have not yet been fully integrated into routine medical practice.

The development journey of a smartphone-based solution for remote assessment of PLwMS will be presented here as a case study to illustrate the design and development process, validation, regulatory and clinical requirements, as well as deployment in the emergent digital health landscape. The process will be discussed from the perspective of industry developers and academic collaborators, from ideation through to technical solution development and version iteration, certification, and deployment. The Floodlight programme is a Roche-led initiative that aims to create digital solutions to facilitate functional assessment in MS. The first Floodlight app was an assessment suite for clinical research that required provisioned smartphones. More recent versions have been developed under design control to ensure that they meet the regulatory standards of reliability and meaningfulness associated with software as a medical device (SaMD)—standalone software that can perform medical functions without being part of a specific medical device hardware [25]—and to enable access for use on personal smartphones in a variety of integrated MS care settings.

## 2. Concept, Proof of Concept, and Assessment of Unmet Needs

The MS digital health space is still largely uncharted. Close partnerships between developers, researchers, HCPs, and PLwMS, from inception and throughout the design process, is essential to ensure that technical solutions, such as smartphone apps, are grounded in science and adequately address unmet patient and/or healthcare needs. Technical development typically begins with the identification and prioritisation of user needs, then the ideation of possible solutions, followed by a “design, test, and iterate” build cycle to ensure those needs are fulfilled. For SaMD development, this creative cycle must also be balanced with clinical, technical, and regulatory processes to ensure the required rigour is achieved. In parallel, it must be established that the solution provides output that is meaningful to both PLwMS and HCPs and that can be readily embedded in the relevant healthcare system.

Technical development for SaMD may begin after proof of concept (PoC) has already been established in a research setting. In the Floodlight PoC study, sensor-based measurement was shown to effectively capture reliable and clinically relevant measures of functional impairment in three domains: cognition, gait, and balance, and hand motor function [26,27]. The Floodlight PoC study constituted sufficient evidence to allow for the use of these assessments in a research setting, but further development under design control was required for deployment in a clinical setting as SaMD. Implementing a secondary, more rigorous technical design step also provided evidence to support face validity and inform features that would facilitate the user experience.

**Figure 1:**
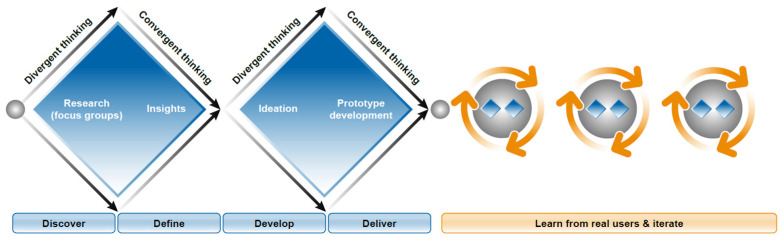
Double Diamond model, an iterative approach with rapid prototyping.

The Double Diamond (DD) model (Figure 1), an iterative approach commonly used in software development, was utilised to guide the process of gaining user insights for the design of the new Floodlight solution. DD is a non-linear model based on divergent–convergent thinking, where a topic is first explored more widely or deeply (divergent) before a focused approach is taken with a singular design solution (convergent) [28]. The iterative aspect of development is then retained through the ongoing acquisition and utilisation of new real-world user insights, experiences, and behaviours to inform subsequent refinement of the solution.

In the initial divergent phase, focus groups with MS experts and PLwMS were conducted to identify the signs and symptoms that might best represent the emergence of progression and to define the current in-clinic standards used to assess functional loss. This process then fed into the convergent phase, which prioritised the assessment of hand motor function, gait, and cognition, all domains frequently affected in PLwMS with worsening disease [29,30,31,32,33,34,35,36,37]. These findings served to substantiate the selection of domain assessments tested in the Floodlight PoC study. During the second divergent phase, exploration of how to technically design the solution and implement the assessments took place, followed by the prioritisation and consolidation of a singular, defined approach for technical development. In order to inform iterative updates and advancements to the solution in the future, mechanisms, such as an analytics platform, were then incorporated to collect insights from real-world users.

**Figure 2:**
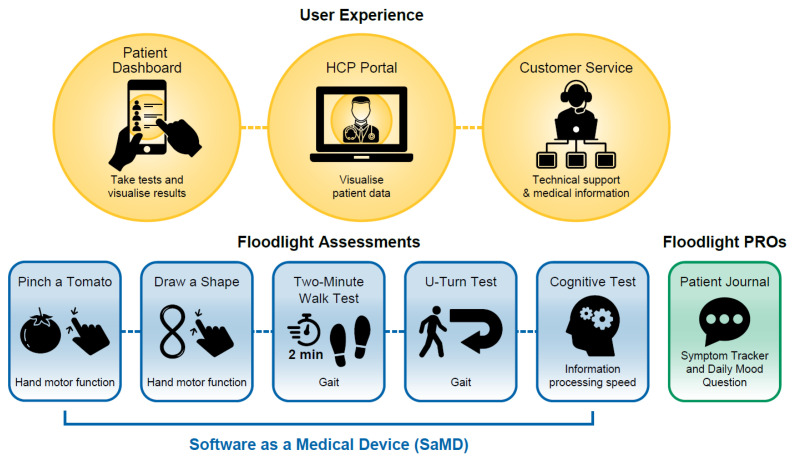
Illustration of current version of Floodlight™ MS v1.2 app and assessments. HCP, healthcare professional; PRO, patient-reported outcome.

This design effort yielded a preliminary structure for the new Floodlight solution, named Floodlight™ MS (currently v1.2). Floodlight MS would provide five assessments for measurement of function across three domains, as well as a Patient Journal (Figure 2). A “bundling approach”, wherein the five assessments would be verified, validated, and independently registered as SaMD, was taken to enable flexibility to change, update, and add new features or assessments without compromising the solution as a whole.

## 3. Desirability: Challenges in Developing a Digital Solution That PLwMS and HCPs Need and Use

Identifying and balancing the needs and desires of different users when creating a digital solution can be challenging. Prior to initiating design control, the Jobs-to-be-Done (JTBD) framework [38] was used to define concrete user need statements for Floodlight MS. JTBD is an outcome-driven innovation strategy used to provide an in-depth understanding of user goals in a structured manner. Core functional desired outcomes (e.g., “Minimize the time it takes to determine how the patient’s past symptoms have changed since their last consultation”), as well as emotional and related jobs that might impact the ability to achieve an outcome (e.g., “Avoid feeling guilty for not spending enough time with a patient”), were collected for each user type. For Floodlight MS solution design, users were defined as (1) individuals with MS who are trying to live their lives while managing their MS, and (2) neurologists who are maintaining MS patients’ quality of life.

JTBD outcome statements reframed the needs related to management of MS into user needs that can be addressed through a technical solution and that can be used to establish parameters for device quality system requirements. To determine which of the needs was most underserved, the desired outcome statements were quantitatively ranked by 202 PLwMS and 211 HCPs in terms of importance of the outcome and current satisfaction in performing the job. For PLwMS, the most underserved needs concerned gaining a better understanding of their health status and treatment management. For HCPs the most underserved needs included monitoring changes in the health status of PLwMS, assessing the impact of MS on daily life, and driving patient compliance. For Floodlight MS, facilitation of improved conversations between PLwMS and their neurologists emerged as a defining priority.

JTBD analysis also clarified factors that might limit the ability of PLwMS to interact with an app, such as comorbidities and disability status. The findings indicated that assessments within the solution must be convenient, with a reasonable duration and frequency. Different levels of user ability in terms of digital skills, as well as aspects such as dexterity and cognitive and visual impairments, would be likely to impact engagement. For many commercial applications, engagement is a key performance indicator and revenue driver. In digital health, however, solutions should only strive for sufficient engagement to support successful outcomes, in order to strike the balance between benefit and burden to the users.

To ensure safety and effectiveness during use, human factors that may affect an individual user’s performance need to be identified and addressed. Errors are frequently caused by the design of the user interface with which users interact. Formative testing with an additional cohort of users is a step in the design control process that serves to identify potential hazardous situations, assess overall usability, and ensure that the interface can be clearly understood and operated per intended use. In formative testing for Floodlight MS, PLwMS expressed satisfaction with an MS-specific solution, which they identified would be a key part of the conversation with their neurologist. PLwMS also indicated that they were more likely to utilise the solution if it were prescribed by an HCP. Formative testing with neurologists was used to assess HCP willingness to adopt the solution and potential barriers to adoption. Neurologists reported that they saw the solution as complementary to their current processes, as long as the data were readily interpretable and easily accessible.

Formative testing informed a significant decision in the developmental journey for the Floodlight programme: the adoption of a prescription-based model for Floodlight MS. This model prioritised partnering with HCPs in a coordinated care setting to identify appropriate patient users, support onboarding and oversee generation and interpretation of patient data. These findings were substantiated by Floodlight Open, a global open-access study, entirely operated via digital interfaces, that was designed to assess adherence to using the app and the feasibility of a “bring your own device” research version of the Floodlight assessment suite provided directly to PLwMS. In line with the adherence issues reported in similar fully digital studies conducted in real-world settings [39], overall adherence in Floodlight Open was low. This contrasts with the controlled environment of the Floodlight PoC study, where good adherence and patient satisfaction were observed [26]. Moreover, in Floodlight Open, adherence rates were positively impacted by concomitant studies that provided clinical coordination. Together, these findings suggested that a supportive clinical care environment would be required to maintain long-term use of the Floodlight MS solution.

Insufficient adherence to remote digital health solutions often presents a challenge to long-term engagement [39]. This is a significant obstacle for developers of apps intended for users with MS, where engagement may be required throughout the user’s lifetime. Adherence to the use of a digital health solution over time may be regarded as a behaviour, determined by factors such as the user’s motivation, ability, and other aspects such as forgetfulness. Behavioural design is based on insights from behavioural science, which can be implemented to aid in evoking desired user behaviour, and is recognised as a key element for development of digital health solutions to increase the likelihood of achieving the desired outcomes [40,41,42]. For example, the concept of the “neurological loop” has been used to explain how habits are formed via a three-step loop composed of cue, routine, and reward; solutions can thus be designed to provide users with strategic rewards to elicit repeated behaviour, based on specific cues [43]. A behavioural design approach was adopted to identify features that might enable users of Floodlight MS to achieve the outcome of improving clinical conversations. Fogg’s behaviour model (Behaviour = Motivation × Ability × Prompt [44]) was used as a framework to audit the design to identify facilitators and barriers to engagement in terms of motivation, ability, and prompts [44,45]. Feedback architecture was then designed to ensure appropriate communication with users and rewards (motivational prompts) for short-, medium-, and long-term outcomes. For example, the PLwMS interface home screen was designed to incorporate prompts for action, a progress indicator, and an appointment calendar to orient use of Floodlight MS around the care conversation. Further, notification and content architecture were also devised to sustain motivation across different use cases.

As there is great variability in symptomology and disease course between individuals living with MS, solutions designed for these users need to accommodate diverse characteristics and varied needs, preferences, and behaviours when utilising smartphones. The complexity of addressing individual preferences and needs in a “one-size-fits-all” approach is typified in the end-user reaction to app gamification. The utility of gamification (the use of game design elements in other contexts) is widely discussed in relation to digital health solution development, as it may aid in increasing motivation and sustaining usage (i.e., increasing adherence [46]); however, any elements need to be applied cautiously in the context of healthcare and must support the desired outcome, which, for Floodlight MS, is the use of data for a care conversation. The topic of gamification—where, in the context of the Floodlight programme experience, some users considered it an inappropriate approach to disease assessment—may represent an example of possible divergent perspectives from different users and user types, which further illustrates the importance of behavioural strategies in studying use patterns. Even after final solution design, regular testing of the applied concepts should be conducted to ensure the usability of the features for all users, aligning with the specific needs of PLwMS. Moreover, careful consideration must be given to how the solution is implemented to ensure effective use. The application of behavioural science, for example through built-in analytics, whilst continuing to develop, test, and iterate on a periodic release cycle, will be important to enable iterative development throughout the solution lifecycle.

## 4. Regulatory Standards: Data Security, Verification and Validation

Challenges and compromises are involved in creating a digital solution that is not only meaningful to end users, but also technically and scientifically robust and aligned with regulatory standards. Digital solutions producing measures adoptable in medical care must meet the standards of device regulatory agencies on design control, cybersecurity and data privacy, risk analysis, and clinical evaluation—all elements considered in the certification of SaMD [25].

**Figure 3:**
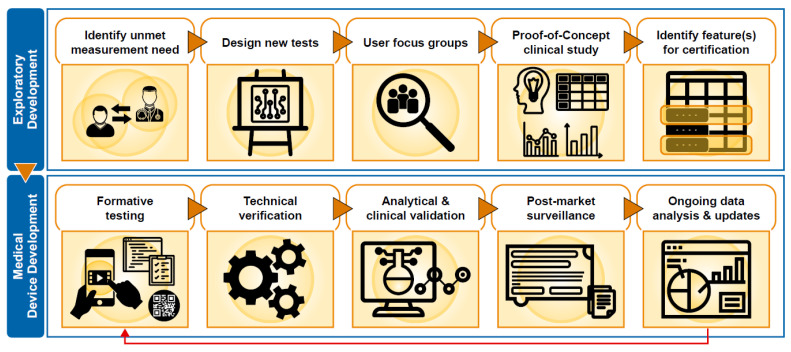
Development of Floodlight™ MS software as a medical device.

To satisfy regulatory requirements, each of the assessments provided by Floodlight MS were subject to technical verification, as well as clinical and analytical validation (Figure 3). Individual assessments and data features were selected for SaMD certification based upon evidence obtained from the Floodlight PoC study and insights from DD.

Technical verification requires assurance that the software is built to the specified requirements. These include elements such as: software unit testing to ensure the functionality of each component, software system testing of new features or components to expose defects in interfaces and interactions between integrated components, and software verification testing to check that the software meets the specified requirements. In modern software development, an agile process is typically implemented to embed quality testing into development, including efforts such as in-sprint level manual testing (in which incremental development and testing occur in tandem) and automation tests to support regression efforts (a software testing practice that ensures an application still functions as expected after any change).

Analytical validation is needed to ensure SaMD output is reliable, accurate, and precise: it demonstrates how well SaMD fulfil their intended use by accurately measuring the desired parameters and generating the correct outputs [47,48]. Analytical validation includes testing the user experience of the solution. This does not require patient or disease-specific assessment, so testing can be conducted in healthy individuals and/or via simulations. For each of the Floodlight MS assessments, robot testing was conducted across 26 mobile devices, representing over 70% of the global smartphone market. Acceptance criteria consisted of three components: observed variability when the same test is repeated on the same smartphone multiple times (within device error), observed variability when the same test is repeated on different smartphones (between device error), and distance between mean measurement of all smartphones to a theoretical ground truth (systemic bias). All smartphones tested passed acceptance criteria with the exception of the Alcatel 7 phone, which failed due to a device chipset issue where screen sizes are not properly reported by the device, rendering the tomatoes in the Floodlight MS “Pinch A Tomato Test” larger than the acceptable range defined in SaMD specifications. All operating systems were also validated. Testing demonstrated that the operating system of the mobile device did not influence data captured. Finally, a series of security tests were conducted, ranging from threat modelling to penetration testing, to ensure data security in the final product.

Whereas analytical validation establishes reliability, a process of clinical evaluation establishes clinical association and validation and is used to determine the sufficiency of evidence and the requirement for further clinical investigation. Clinical evaluation is a systematic and methodologically sound process used to continuously generate, collect, analyse, and assess the clinical data pertaining to a device in order to verify and validate the safety and performance of the device, including any clinical benefits, in the target user population and when used as intended [49]. Each assessment in the Floodlight MS solution was subject to evaluation, supported by multiple evidence sources, including the Floodlight PoC study and an observational study with PLwMS that provided an assessment by clinical content area experts that the process of, and results from, the Floodlight MS assessments achieved their intended purpose.

Post-marketing surveillance of SaMD is also required to provide ongoing monitoring of any defects and/or safety concerns, in order to ensure that solutions are safe and effective during real-world use. The post-market clinical follow-up plan, which is part of the clinical evaluation, specifies methods and procedures for collection and evaluation of clinical data from on-market use to confirm safety and performance. In addition to implementing subsequent clinical trials and real-world evidence generation initiatives, customer support should be present to capture reportable SaMD events for investigation, such as technical defects and safety issues, in order to fully comply with regulatory requirements. For Floodlight MS, customer support was tailored to respond to users’ needs (HCPs and PLwMS) in a specific geography, for example by offering local language support, in addition to addressing technical and medical questions.

Significant effort from the developer is required to achieve robust, regulatory-grade clinical validation. Given the rapidly evolving nature of digital technology, it is also critical for developers to find effective approaches to continuously advance solution design. Likewise, continual technological advancement presents a challenge to regulators, who must concurrently advance policy in order to foster growth of digital innovation for better disease management [17]. To this end, regulatory agencies are actively facilitating collaborative initiatives within the digital community to advance digital health innovation [50].

Two key aspects of this dialogue are data safety and cybersecurity, as it is crucial to not only establish and maintain robust data privacy and security, but also to adapt them to comply with local requirements across geographies (e.g., General Data Protection Regulation in the EU, the Health Insurance Portability and Accountability Act in the USA, etc.). Data security can be divided into technical (obtaining and storage of data), methodological (the software application and infrastructure used to deliver it), and procedural aspects (data usage, data access, and security breaches), and each of these must be carefully considered at each stage of design and development [51]. Demonstrating a robust approach to personal data security is also a means to build users’ trust: 45% of users worry about the unwanted use of their data when using mobile devices for health-related activities [52,53], and there are legitimate concerns over user identification, data sharing with third parties, or accidental data leakage [52]. As data privacy and security provisions must be placed at the fore from the start of a user’s interaction with a digital solution, Floodlight MS users are presented with a data privacy notice during the sign-up process. This contains detailed information on the treatment of their personal information, which is in line with the applicable regulatory frameworks. Different types of security measures are in place for Floodlight MS, including password-protected access with automatic logout after a period of inactivity, and data encryption in transit and at rest. Moreover, the legal manufacturer of the device ensures that appropriate training and processes for data management operators are set up and that action plans are ready in case of any incidents.

After regulatory clearance has been achieved, the hurdles of adoption into medical practice, making the solution accessible, and providing ongoing monitoring and managing the system need to be overcome. All these aspects will involve the collaborative efforts of multiple healthcare stakeholders such as PLwMS, HCPs, and payers, as these hurdles cannot be overcome without participation from all relevant parties [17].

## 5. Taking an Adaptive Approach

Agility is key when developing digital solutions, requiring a fluid approach to facilitate an iterative developmental process that aligns with the design control and requisite regulatory requirements. The rapidly changing technological environment contrasts markedly with classical drug development with its careful, largely linear and standardised processes [54,55]. Once a new solution is developed and deployed, post-market data can serve to further validate clinical effectiveness, evolve technical capabilities, and refine the user experience, and may even support subsequent regulatory engagement and reassessment.

Real-world evidence generation and non-interventional studies are often more efficient than, and can be complementary to, interventional trials. For the Floodlight programme, non-interventional studies and real-world evidence generated with Floodlight MS serves to complement assessment of Floodlight test technology in more formal clinical trial settings. Research is also being conducted to improve the clinical utility of the test suite, support clinical analysis, develop quality control features, and advance the understanding of sensor data [56,57,58,59].

The development path outlined here culminated in the release of Floodlight MS, which contains the five assessments registered as SaMD. Additional features, such as the Patient Journal, designed to help users reach the outcome of improved care conversations, are grouped separately and are thus able to be more frequently and flexibly iterated and improved upon based on user feedback, without necessitating resubmission to regulatory authorities. This continual iteration is enabled by recurring development cycles, which allow improvements to be implemented frequently, generating updated versions several times throughout the year. Floodlight MS is set to continue evolving, and thus certain topics covered in this paper may be revisited as knowledge advances.

The use of different technical deployment models, tailoring the means by which the solution is provided, may also provide the flexibility needed to meet local regulatory requirements and enable interoperability and integration in a complex and fragmented electronic health records landscape [60,61]. Three deployment models were designed for Floodlight MS: (i) a standalone solution with an HCP-facing web-based portal for data access, (ii) a standalone solution that can be integrated with electronic health records, and (iii) a software development kit (SDK). The SDK enables rapid, tailored integration of the Floodlight MS assessments into a third-party solution, for example into the DreaMS digital research tool advanced by the Research Center for Clinical Neuroimmunology and Neuroscience, Basel (RC2NB) [62]. The SDK approach also allows for integration into solutions developed in-house. Tailoring the deployment model for a digital solution may enable greater interoperability and the capability to address diverse and local needs on a greater scale.

## 6. Future Horizons

The provision and adoption of technological solutions and the sharing of information globally has the potential to drive knowledge acquisition and positively affect healthcare worldwide [63]. Digital solutions offer great promise in delivering increasingly individualised, easily accessible, and effective healthcare, with the capacity to evolve with time and adapt to the changing needs of PLwMS and HCPs. The impact of the COVID-19 pandemic has given additional proof of such versatility and usefulness, highlighting how barriers can be overcome through the adoption of digital tools [64], where capturing digital data remotely may mean that symptom tracking can be maintained even when clinic visits are not possible. Ultimately, digital solutions must contribute to the long-term resolution of broader health system challenges, such as lack of access to care, lack of frequent monitoring, costly and ineffective treatment, and delayed diagnosis of MS disease progression.

Fundamentally, digital solutions such as Floodlight MS aim to improve outcomes for PLwMS. To reach this objective, continued collaboration and partnership with the entire MS community is needed, not only to continually refine individual solutions but also to create robust standards for implementation, interpretation, and interoperability. Ongoing investment into the clinical development of a digital solution will also enable continuous improvement, enhancing the clinical utility and sustained actionability of a given solution. This is especially relevant for solutions such as Floodlight MS, which generate data that may be used to improve our understanding of the disease or create digital biomarkers. The use of such data could serve to bridge the gap between clinical trials and medical care, for example, by enabling the creation of a baseline dataset in routine care prior to clinical trial enrolment; or by enabling a more immediate comparison of population outcomes to individual performance using the same reliable, objective outcome measures.

The sharing and secondary use of the data collected using digital solutions will be important for shaping the future of research, regulation, and policymaking in the digital healthcare sphere. Platforms such as the European Health Data Space are being developed to facilitate data sharing across sectors [65]. Key areas centre around health data exchange, access to health data for research and policymaking, and a single market for digital health products and services. Structuring projects to generate data for secondary use in collaboration with the scientific community will help to shape the digital space by furthering research, as well as from a regulatory perspective in terms of enabling better fit-for-purpose and evidence-based policymaking.

The Floodlight programme has undertaken a path of SaMD design and development to support safe and effective use of Floodlight MS, a bundled digital assessment solution for PLwMS. The incorporation of design strategies commonly used in software development informed features to support user adherence, clinical utility, and readiness for integration into today’s fragmented global healthcare landscape. This effort is underpinned by an iterative and collaborative clinical validation, technical refinement, and deployment approach intended to drive continual evolution of the technology. Ultimately, the emergence of robust digital solutions may help to change the way that disease progression is measured in MS, enabling optimisation of care, and helping to bridge clinical trial and medical practice data.
